# Balanced bifrontal transcranial direct current stimulation enhances working memory in adults with high-functioning autism: a sham-controlled crossover study

**DOI:** 10.1186/s13229-017-0152-x

**Published:** 2017-07-28

**Authors:** J. Jason van Steenburgh, Mark Varvaris, David J. Schretlen, Tracy D. Vannorsdall, Barry Gordon

**Affiliations:** 1 Department of Neurology, The Johns Hopkins University School of Medicine, 1629 Thames Street, Suite 350, Baltimore, MD 21231 USA; 2 Department of Psychiatry and Behavioral Sciences, The Johns Hopkins University School of Medicine, 600 N. Wolfe Street, Baltimore, MD 21287 USA; 3 Division of MR Research, Russell H. Morgan Department of Radiology and Radiological Science, 600 N. Wolfe Street, Baltimore, MD 21287 USA; 4 Department of Neurology, The Johns Hopkins University School of Medicine, 600 N. Wolfe Street, Baltimore, MD 21287 USA

**Keywords:** Autism, Working memory, Transcranial direct current stimulation, Dorsolateral prefrontal cortex

## Abstract

**Background:**

Working memory (WM) often is impaired in autism spectrum disorder (ASD). Such impairment may underlie core deficits in cognition and social functioning. Transcranial direct current stimulation (tDCS) has been shown to enhance WM in both healthy adults and clinical populations, but its efficacy in ASD is unknown. We predicted that bifrontal tDCS would improve WM performances of adults with high-functioning autism during active stimulation compared to sham stimulation and that such enhancement would generalize to an untrained task.

**Methods:**

Twelve adults with high-functioning ASD engaged in a battery of WM tasks that included backward spatial span, backward digit span, spatial *n*-back and letter *n*-back. While engaged, 40 min of 1.5 mA bifrontal stimulation was applied over the left and the right dorsolateral prefrontal cortices (DLPFC). Using a single-blind crossover design, each participant received left anodal/right cathodal stimulation, right anodal/left cathodal stimulation, or sham stimulation, in randomized counterbalanced order on three separate days. Following tDCS, participants again engaged in letter and spatial *n*-back tasks before taking the Brief Test of Attention (BTA). We used repeated-measures ANOVA to compare overall performance on the WM battery as measured by a composite of *z*-scores for all five measures. Post hoc ANOVAs, *t* tests, Friedman’s tests, and Wilcoxon signed-rank tests were used to measure the online and offline effects of tDCS and to assess performances on individual measures.

**Results:**

Compared to sham stimulation, both left DLPFC anodal stimulation (*t*_11_ = 5.4, *p* = 0.0002) and right DLPFC anodal stimulation (*t*_11_ = 3.57, *p* = 0.004) improved overall WM performance. Left anodal stimulation (*t*_11_ = 3.9, *p* = 0.003) and right anodal stimulation (*t*_11_ = 2.7, *p* = 0.019) enhanced performances during stimulation. Enhancement transferred to an untrained task 50 min after right anodal stimulation *(z*_11_ = 2.263, *p* = 0.024). The tasks that showed the largest effects of active stimulation were spatial span backward (*z*_11_ = 2.39, *p* = 0.017) and BTA (*z*_11_ = 2.263, *p* = 0.024).

**Conclusions:**

In adults with high-functioning ASD, active bifrontal tDCS given during WM tasks appears to improve performance. TDCS benefits also transferred to an untrained task completed shortly after stimulation. These results suggest that tDCS can improve WM task performance and could reduce some core deficits of autism.

**Trial registration:**

NCT01602263

## Background

Many individuals with autism spectrum disorder (ASD) show impairments in working memory (WM) [1–3]. Working memory refers to the capacity to maintain, update, and manipulate information held in temporary storage [4]. Poor WM performance has been shown in children and adolescents with ASD [5–10] and in adults with ASD [11–13]. Although other executive function deficits can resolve somewhat with age, WM deficits tend to persist [3, 14].

Working memory is critical for many complex cognitive functions including language [15, 16], general intelligence, and reasoning [17]; therefore, WM deficits likely produce profound effects in individuals with autism. Poor WM likely contributes to social problems in people with ASD [18] because maintaining continually changing social information in temporary storage (WM) is necessary for social flexibility [19]. Working memory also drives the ability to encode emotions observed on faces [20], regulate emotional responses [21], and break from restrictive or repetitive behaviors [22]. Therefore, remediating WM deficits could improve some of the core cognitive and behavioral deficits that characterize ASD.

Unfortunately, behavioral approaches to WM remediation have had limited effects [23], and attempted behavioral interventions have suffered from high attrition rates [24]. It also remains unclear whether improvements that may occur from behavioral remediation would generalize to other tasks or abilities [25]. Therefore, a simpler and faster approach to improving WM deficits might be more useful.

Transcranial direct current stimulation (tDCS) is a non-invasive form of brain stimulation accomplished by the passage of a weak electrical current, typically in the range of 0.5–2.0 mA, through the scalp and skull via surface electrodes. The bases for tDCS effects have not been conclusively determined, but tDCS may modulate neural activity and behavior via several mechanisms, including alteration of membrane potentials [26], direct action at synaptic receptors [27], and downstream effects on network plasticity [28].

When applied over the dorsolateral prefrontal cortex (DLPFC), tDCS has enhanced verbal WM in healthy adults [29–34] and in patients with schizophrenia [35, 36], Parkinson’s disease [37], stroke [38], aphasia [39], and depression [40–42]. Anodal tDCS applied to the left prefrontal cortex in healthy adults has been shown to increase prefrontal cortex functional connectivity and to strengthen bilateral fronto-parietal networks [28]. Anodal tDCS to the right prefrontal cortex also has been shown to strengthen ipsilateral fronto-parietal connectivity and disrupt default mode network integrity [43], which potentially could be among the mechanisms for tDCS effects on WM.

Adults with ASD show prefrontal hypoactivation during working memory tasks [13, 44]. They also show reduced anterior-posterior connectivity [44–46]. Because WM depends on prefrontal activity [47] and communication between the DLPFC and posterior parietal resources [48], such deficits may provide at least a partial explanation for poor WM performance in individuals with ASD [49].

Therefore, there are both empirical and theoretical reasons for our primary aim, which is to determine whether tDCS applied over the prefrontal cortices improves WM in individuals with autism. The choice to use balanced bifrontal stimulation (F3-F4), rather than the more typical F3-right supraorbital montage or a unilateral montage, was motivated by several considerations. First, imaging and lesion studies suggest that WM is domain general [17, 50] and bilateral prefrontal resources are recruited for many WM tasks [51, 52]. Second, cathodal stimulation’s inhibitory effects on cognition are often weak or nonexistent [53, 54]; and in tasks that depend on bilateral resources, such effects may be countered by the effects of contralateral disinhibition. Third, balanced bilateral stimulation seems to distribute current more deeply [55] and broadly [56] and enhance functional connectivity more than what would be expected from the summed effects of the two electrodes due to interhemispheric interactions [57], which might contribute to a greater effect on widely distributed WM networks. Finally, when directly compared to unilateral montages or unbalanced bilateral montages, balanced bilateral stimulation (dual-hemisphere stimulation of homologous structures) has been shown to more effectively enhance motor learning [58, 59] and improve tactile discrimination [60]. Although no published studies or meta-analyses have directly compared the effectiveness of balanced bifrontal montages with unilateral or unbalanced bifrontal montages in the enhancement of WM, Richmond and colleagues [33] referred to pilot studies that showed that an F3-F4 montage was more effective than a F3-right supraorbital area montage. Balanced bifrontal montages have been shown to enhance WM accuracy [33, 35, 39–42, 56] with small to medium effect sizes in healthy adults [33, 56] and medium to large effect sizes in clinical populations [35, 39, 40, 42]. Data reported in meta-analyses suggest that unbalanced frontal stimulation (usually DLPFC-contralateral supraorbital area) tends to have a small effect on WM accuracy in both healthy populations [61] and clinical populations [62].

We explored two different active stimulation conditions because it is unknown whether the left or right DLPFC is a more effective target site for working memory enhancement in individuals with ASD. WM is not a monolithic mental function, but many of its sub-processes are mediated by activity in the lateral prefrontal cortex [63, 64]. Therefore, we used a short battery of WM tasks that require varying degrees of information maintenance and manipulation, and we used stimuli from different modalities. We explored an intervention in adults with ASD because WM deficits persist in ASD [3, 14] and because adults typically have aged out of behavioral modification programs and are developmentally stable.

We predicted that (1) applying active tDCS during WM training would enhance WM performance compared to sham stimulation, (2) material specificity of effects would not depend on the hemisphere stimulated by the anode, and (3) after stimulation, enhancement would transfer to an untrained task with a working memory component. We used composite *z*-scores as our primary outcome to capture overall WM enhancement across stimulus modalities.

## Methods

### Participants

The Institutional Review Board of The Johns Hopkins School of Medicine approved the study, and all participants gave written informed consent to participate. Twelve adults who were previously diagnosed with ASD were recruited from a Baltimore area residential and day center for adults with ASD. Their ages ranged from 20 to 66 years (*M* = 32.1; SD 12.4). They included 10 men and 2 women of whom 10 were Caucasian, 1 was African-American, and 1 was Asian. All participants had acquired language during childhood and spoke English as their first language. Potential subjects were excluded if their records or subsequent interviews suggested a history of neurological disease, psychiatric disorder, or active use of antipsychotic medications. The participants had IQ scores that ranged from 71 to 144 (*M* = 100.1; SD 23.1) on the Kaufman Brief Intelligence Test [65] and thus were classified as high functioning [1]. They had completed 12–21 years of schooling (*M* = 14.3; SD 3.1). Each study participant underwent a baseline assessment with the Autism Diagnosis Observation Schedule (ADOS) Module 4 [66], and the group was found to have a mean communication + social interaction score of 12.2 (SD 3.0), with scores of 4.2 (SD 1.4) on communication and 8 (SD 2.1) on social interaction. All participants met the ADOS cutoff of 7 for a diagnosis of ASD. All behavioral data were collected in a quiet room at The Johns Hopkins Hospital.

### Test materials and tasks

#### Letter and spatial *n*-back

**Fig. 1:**
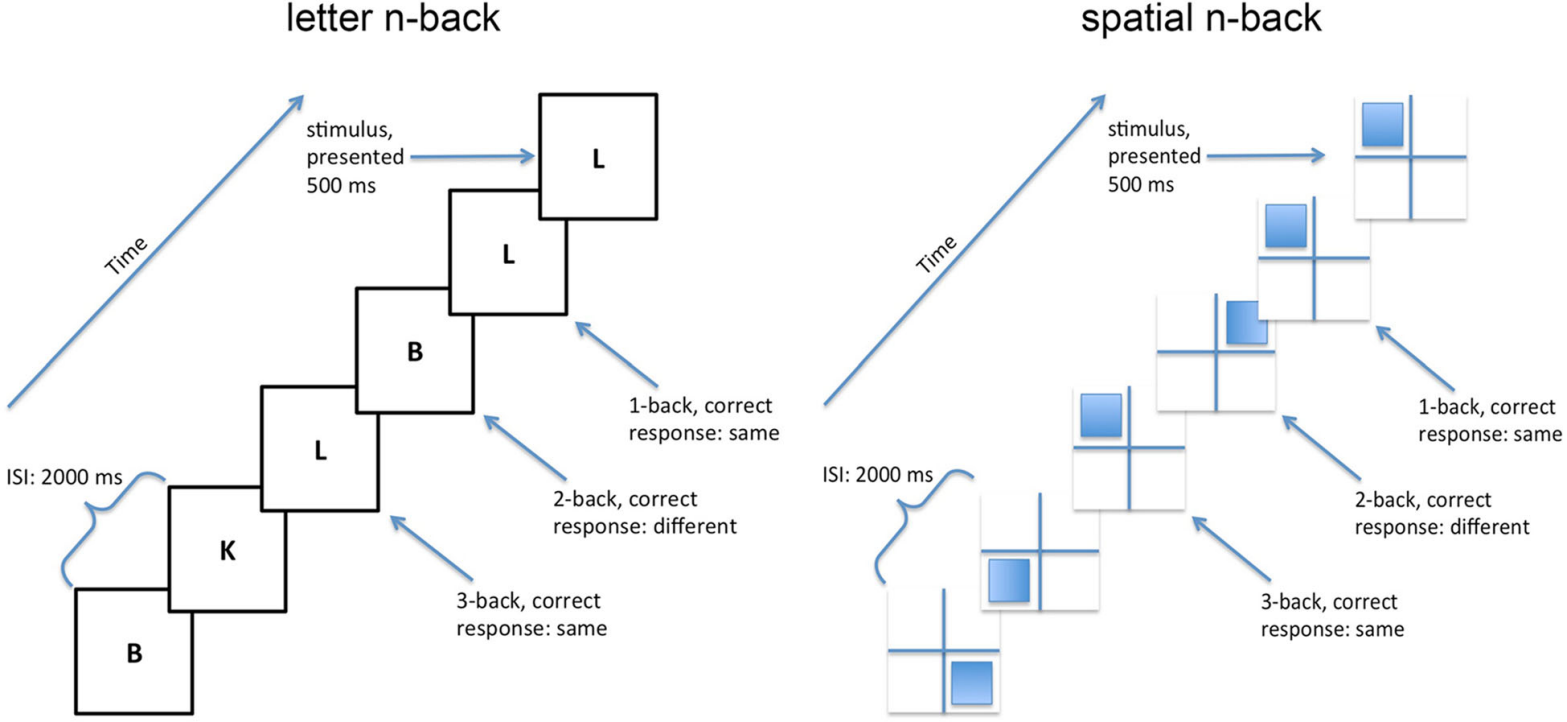
Letter and spatial *n*-back task procedures

The *n*-back stimuli were displayed using E-Prime E Studio (v2.0 Psychology Software Tools, Inc.) on a Dell Inspiron N5110 with a 15.6-cm screen (diagonal length). Letter *n*-back stimuli were pseudo-randomly chosen from among the 20 consonants of the English alphabet. Letters were presented in the middle of the screen (10% screen height) in black bold Helvetica on a white background. During spatial *n*-back, a constant 1-cm thick blue fixation cross was extended to the full width and height of the screen. Blue rectangles appeared pseudo-randomly in one of the four quadrants, covering 75% of the available space in the quadrant. For both letter and spatial *n*-back tasks, stimuli were displayed for 500 ms with a 2000-ms inter-stimulus interval. Participants indicated whether the stimuli were the same or different than the previous stimuli by pressing buttons on the keyboard. If participants did not respond within 2500 ms after stimulus onset, a fixation cross appeared, followed by the next stimulus. The load for each block increased from 1-back to 2-back to 3-back. At the top of the screen, a reminder message identified the current task load, e.g., 2-back. Brief visual instructions were presented prior to every change in load to show what was meant by 1-back, 2-back, or 3-back. All participants completed 3-minute practice blocks that included 12 trials at each load at the beginning of the stimulation period. On the practice trials, response accuracy feedback was provided: The screen flashed green for 50 ms for a correct response or red for an error or failure to respond within 2500 ms. Participants were told that failure to respond would be counted as incorrect. During stimulation, each participant completed one 12-min block of 198 spatial *n*-back trials and one 12-min block of 198 letter *n*-back trials (each block consisting of 66 trials at loads of 1-, 2-, and 3-back) to assess the online effects of tDCS. Following the cessation of stimulation, each participant also completed two 6-min blocks of 102 trials (each with 34 trials at loads of 1-, 2-, and 3-back) to assess the offline effects of tDCS on WM. *N*-back accuracy was calculated as (hits + correct negatives)/(total items). The *n*-back tasks are depicted in Fig. 1.

#### Wechsler Memory Scale, 3rd Edition (WMS-III)

The WMS-III digit span backward and spatial span backward tasks were administered as per the test manual [67]. Briefly, participants repeated strings of digits in reverse order for digit span backward and tapped a sequence of blocks on a board in reverse order for spatial span backward. Forward digit span and forward spatial span tasks were omitted.

#### The Brief Test of Attention (BTA)

The BTA was given as per the instruction manual [68] except that each participant received only one form (i.e., either numbers or letters) as determined by computer-generated random number assignment. Participants listened to 10 strings of letters and numbers (e.g., F-3-7-R-4-2-Q) and were required to ignore the numbers (or letters) and to continually rely on working memory to update a tally of how many letters (or numbers) were read aloud in each string. The raw score is the total number of correctly tallied strings, ranging from 0 to 10. All items in the subtest were administered regardless of performance.

#### Stimulation side-effect questionnaire

On a 16-item side effects questionnaire that we developed based on references to side effects in the literature [69–71], participants rated their experiences of headache, difficulty with concentration, discomfort, fatigue, pain, tingling, nausea, anxiety, anger, confusion, happiness, sadness, tension, fear, alertness, and vision changes. Participants circled a number on a Likert scale ranging from 0 to 10 (0 = absent; 10 = most extreme). Subjects were given this questionnaire before and after each tDCS session. At the end of the questionnaire given after each session, the participants were asked to indicate (yes/no) whether they thought that they had just received real stimulation [72].

### tDCS stimulation parameters

Transcranial direct current stimulation was delivered by a neuroConn DC-stimulator PLUS (http://www.neurocaregroup.com/dc_stimulator_plus.html). In all conditions, two flat carbon rubber electrodes (surface area 25 cm^2^) encased in saline-soaked sponges were placed over F3 and F4 using the 10–20 international electrode positioning system. The electrodes were secured evenly over the scalp with 1-in. wide rubber straps: One strap was parallel to the horizontal plane and the second strap was anchored to the first strap and arched over F3 and F4. Electrode sponges were aligned so that the bottom sides were parallel with the strap wrapped around the horizontal plane. The active stimulation conditions were designated according to the location of the anodal electrode for the sake of brevity, not to imply that the anode was more important than the cathode or that their effects would be distinguished from one another. There were three conditions:

1. Left anodal: The anode was over F3 and the cathode over F4. Stimulation was ramped up from 0 to 1.5 mA on a sinus curve for 15 s, held constant at 1.5 mA (current density = 0.06 mA/cm^2^) for 39.5 min, and ramped back down to 0 mA on a sinus curve for 15 s.
2. Right anodal: The anode was over F4 and the cathode over F3. Stimulation was ramped up from 0 to 1.5 mA on a sinus curve for 15 s, held constant at 1.5 mA (current density = 0.06 mA/cm^2^) for 39.5 min, and ramped back down to 0 mA on a sinus curve for 15 s.
3. Sham: The anode was over F3 and the cathode over F4. Stimulation was ramped up from 0 to 1.5 mA on a sinus curve for 15 s, held constant at 1.5 mA for 30 s, and ramped back down to 0 mA on a sinus curve for 15 s.

### Procedures

**Fig. 2:**
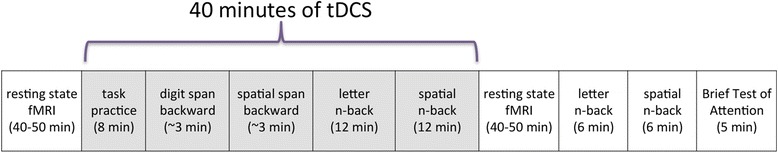
Experimental protocol. All participants completed the same protocol on three separate days. They received either left anodal stimulation, right anodal stimulation, or sham stimulation in counterbalanced order

The sequence of procedures is indicated in Fig. 2.

In a 3-session crossover design, participants were randomly assigned via computerized random number generator to one of six groups with order of condition counterbalanced (left anodal, right anodal, sham). Group sizes were evenly balanced. Participants were given the KBIT-2 [65] prior to session 1. Before each tDCS session, they were scanned in a Siemens 3 T MRI for 13 min (results not reported here, but see [73]). Thereafter, the tDCS device was affixed and participants completed the side-effects questionnaire prior to stimulation. During stimulation, participants engaged in 8 min of test explanations and practice, followed by digit span backward and spatial span backward tasks, 12 min of letter *n*-back problems, and 12 min of spatial *n*-back problems. After the 40-min period of active or sham stimulation ended, participants again completed the side-effects questionnaire and the tDCS apparatus was removed. Then, following an approximately 40-min interval during which resting-state fMRI data again were collected, participants completed the offline *n*-back tasks, as described above, followed by the BTA. Other than stimulation parameters, the protocol was identical in all three sessions. Sessions were separated by a mean of 6.8 (5.1) days, with a minimum washout of 24 h between sessions. Testing was single blind; the participants were not aware of the test conditions, although the technicians were.

### Analyses

**Fig. 3:**
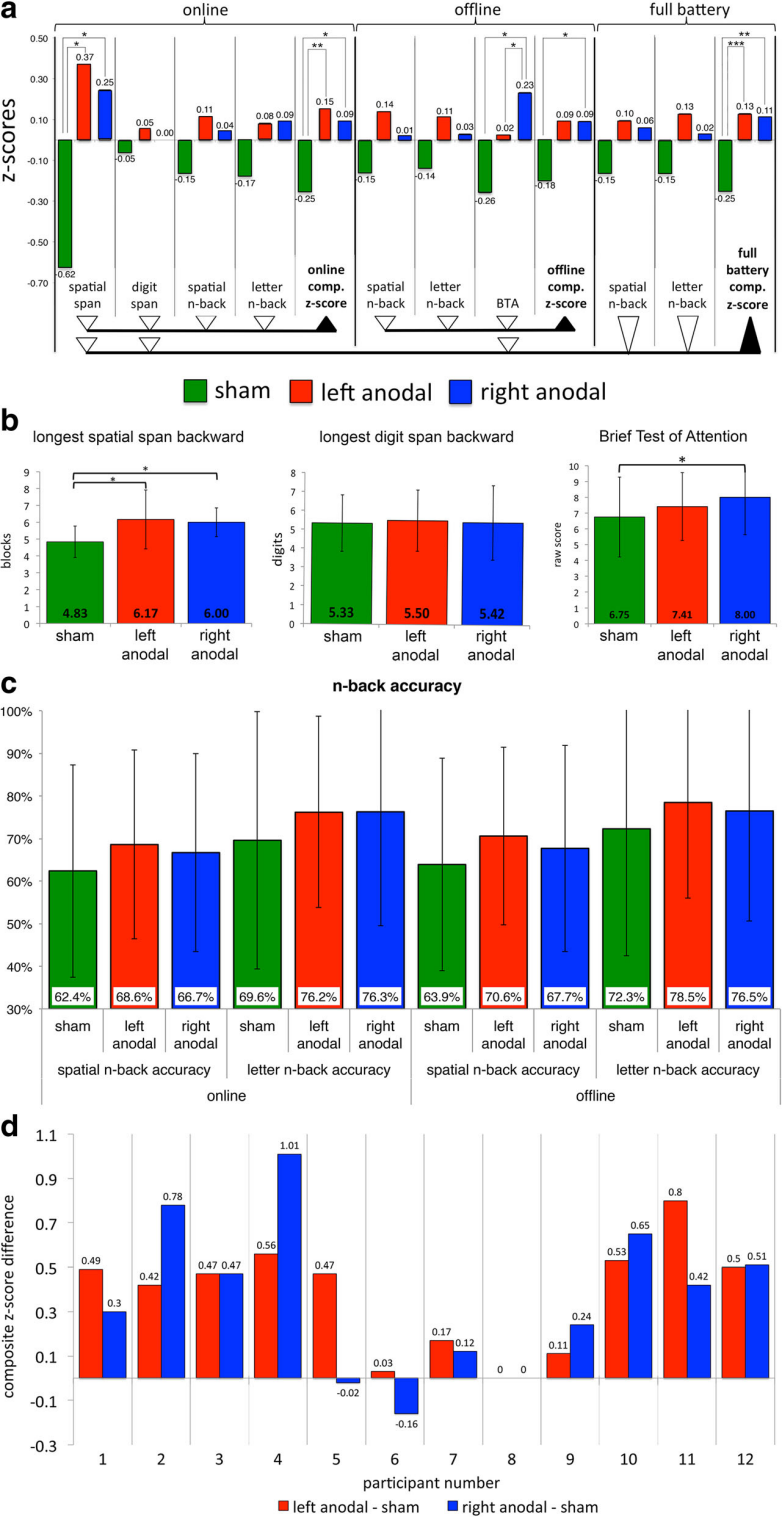
**a** *z*-scores for individual tests and composite *z*-scores. Composite *z*-score (online) is the mean of four *z*-scores: spatial span backward maximum length, digit span backward maximum length, online letter *n*-back accuracy, and online spatial *n*-back accuracy. Composite *z*-score (offline) is the mean of three *z*-scores: offline letter *n*-back accuracy, offline spatial *n*-back accuracy, and BTA raw score. Composite *z*-score (full battery) is the mean of five *z*-scores: spatial span backward maximum length, digit span backward maximum length, letter *n*-back accuracy (mean of online and offline percentages), spatial *n*-back accuracy (mean of online and offline percentages), and BTA raw score (*p < 0.05, **p < 0.01, ***p < 0.001). **b** Backward spatial span, backward digit span, and Brief Test of Attention task results. *Error bars* indicate standard deviations. (*p < .05). **c** *N*-back accuracy. Percentage correct for online spatial *n*-back, online letter *n*-back, offline spatial *n*-back, and offline letter *n*-back. *Error bars* indicate standard deviations. **d** Individual differences in full-battery composite *z*-scores (vs. sham) for left anodal and right anodal stimulation. Mean composite *z*-scores (full battery) were −0.25 (SD 0.71) for sham stimulation, 0.13 (SD 0.82) for left anodal stimulation, and 0.11 (SD 0.81) for right anodal stimulation

With a one-way repeated measures design, we tested our hypothesis that tDCS would enhance overall WM performance during and after active stimulation compared to sham stimulation by examining performance on a cognitive test battery. As our primary outcome measures, we computed composite *z*-scores for each participant. One reason that composite *z*-scores were used in the primary analysis was because the number of participants was suboptimal with regard to power, and the participants were anticipated to vary widely in IQ and education. Composite *z*-scores are an effective tool to evaluate performance on a battery of tests that recruit resources from a common cognitive domain [74–76] because they increase statistical power by attenuating floor and ceiling effects, they reduce random variability, and they lower the number of correlated data in analyses [77]. Participants’ raw scores were transformed to *z*-scores using all 36 data points (12 participants × 3 sessions) obtained for each test. The full-battery composite *z*-score was the mean of the participant’s *z*-scores for all five working memory tests: WMS-III spatial span backward (longest length), WMS-III digit span backward (longest length), verbal *n*-back accuracy (mean of online and offline scores), spatial *n*-back accuracy (mean of online and offline scores), and BTA (raw score). We also computed an online composite *z*-score for the four online measures of working memory and an offline composite *z*-score for the three offline measures (see Figs. 2 and 3e), based on the fact that BTA performance requires a significant working memory component (updating). Measures of working memory capacity and accuracy were chosen, rather than reaction time, because those measures have been correlated with behaviors related to core deficits in autism [20–22] and because tDCS tends to enhance accuracy more than reaction time in clinical populations [78]. The main effect of stimulation on overall WM performance (full-battery composite *z*-score) was analyzed with repeated-measures ANOVA, with stimulation condition as the independent factor, followed by post hoc, paired-sample *t* tests. We compared the online composite *z*-scores across conditions with repeated measures ANOVA and post hoc, paired-sample *t* tests. We compared the offline composite *z*-scores across conditions with Friedman’s and Wilcoxon signed-rank tests because the data were not normally distributed (Shapiro-Wilk, *p* = 0.045). For secondary analyses, we examined the effects of active vs. sham stimulation on individual WM measures in native space using repeated measures ANOVA and post hoc *t* tests or with Friedman’s and Wilcoxon signed-rank tests. We also tested the effectiveness of blinding study participants to the stimulation conditions as measured by self-report. All analyses were conducted using the Statistical Package for the Social Sciences (SPSS) for Windows, Version 22.0 (SPSS Inc., Chicago, IL, USA). Effect sizes for paired-sample *t* tests were Cohen’s *d*_*s*_ = mean difference/pooled SD. Effect sizes for Wilcoxon signed-rank tests were *r* = *z*/√(2*N). For the purpose of comparing the effects reported here to those in other studies, we provided Cohen’s *d*_*s*_ effect sizes for all significant analyses.

## Results

### Primary outcome: overall WM performance

As shown in the final row of Table 1, the composite *z*-scores (full battery) were −0.25 (SD 0.71) for sham stimulation, 0.13 (SD 0.82) for left anodal stimulation, and 0.11 (SD 0.81) for right anodal stimulation, which repeated measures ANOVA showed to be significantly different (*F*_2,22_ = 12.85, *p* = 0.0002). Post hoc, paired-sample *t* tests revealed that performances associated with both left anodal active stimulation (*t*_11_ = 5.4, *p* = 0.0002) and right anodal active stimulation (*t*_11_ = 3.57, *p* = 0.004) were better than performances associated with sham tDCS. For composite *z*-scores (full battery), Cohen’s *d*_*s*_ effect sizes were 0.50 for left anodal stimulation and 0.47 for right anodal stimulation. There was no difference in overall WM performance between the two active stimulation conditions (*t*_11_ = 0.26, *p* = 0.796). To assess the role of session order on full-battery composite *z*-scores, we compared them across sessions. We found composite *z*-scores (full battery) of −0.03 (SD 0.81) for session 1, −0.06 (SD 0.75) for session 2, and 0.09 (SD 0.83) for session 3. Repeated measures ANOVA showed no significant practice effects across sessions (F_2,22_ = 0.88, *p* = 0.428).

**Table 1:** Behavioral data and statistics: Composite *z*-score (online) is the mean of four *z*-scores: spatial span backward maximum length, digit span backward maximum length, online letter *n*-back accuracy, and online spatial *n*-back accuracy. Composite *z*-score (offline) is the mean of three *z*-scores: offline letter *n*-back accuracy, offline spatial *n*-back accuracy, and BTA raw score. Composite *z*-score (full battery) is the mean of five *z*-scores: spatial span backward maximum length, digit span backward maximum length, letter *n*-back accuracy (mean of online and offline percentages), spatial *n*-back accuracy (mean of online and offline percentages), and BTA raw score. Also included are means and standard deviations for 1-back, 2-back, and 3-back for letter and spatial *n*-back, both online and offline

	Sham	Left anodal	Right anodal	Repeated measures ANOVA F(2,22)	Friedman’s Test χ2	*p* value
WMS-III, spatial span backward, longest span (blocks)	4.8 (0.9)	6.2 (1.8)	6.0 (0.9)		9.24	0.01
WMS-III, digit span backward, longest span (digits)	5.3 (1.5)	5.5 (1.6)	5.4 (2.0)	0.12		0.891
Spatial *n*-back accuracy (online)	62.4% (25.0%)	68.6% (22.1%)	66.7% (23.2%)		3.17	0.205
1-back	74.7% (23.1%)	80.0% (19.9%)	79.6% (21.4%)			
2-back	61.4% (27.9%)	68.5% (26.4%)	66.2% (25.5%)			
3-back	49.7% (25.8%)	55.6% (22.9%)	52.7% (25.0%)			
Letter *n*-back accuracy (online)	69.6% (30.2%)	76.2% (22.5%)	76.3% (26.8%)		0.5	0.779
1-back	77.3% (26.6%)	82.9% (19.9%)	84.8% (21.7%)			
2-back	70.4% (32.4%)	76.0% (26.0%)	76.2% (29.3%)			
3-back	61.1% (33.1%)	69.7% (24.2%)	68.1% (29.7%)			
Composite *z*-score (online)	−0.25 (0.74)	0.15 (0.87)	0.09 (0.81)	7.68		0.003
Spatial *n*-back accuracy (offline)	63.9% (24.9%)	70.6% (20.9%)	67.7% (24.2)	0.55		0.758
1-back	76.2% (21.9%)	85.1% (20.2%)	78.4% (21.7%)			
2-back	63.2% (29.0%)	70.5% (24.3%)	69.8% (26.8%)			
3-back	52.5% (26.5%)	56.3% (21.8%)	54.6% (26.0)			
Letter *n*-back accuracy (offline)	72.3% (29.8%)	78.5% (22.5%)	76.5% (25.9%)		0.17	0.92
1-back	80.7% (23.2%)	86.1% (20.7%)	84.3% (21.6%)			
2-back	71.1% (33.2%)	77.8% (22.3%)	77.8% (27.2%)			
3-back	65.1% (33.6%)	71.6% (26.2%)	67.4% (30.0)			
Brief Test of Attention (offline)	6.8 (2.5)	7.4 (2.2)	7.9 (2.5)		7.09	0.029
Composite *z*-score (offline)	−0.18 (0.92)	0.09 (0.77)	0.09 (0.93)		7.17	0.028
Spatial *n*-back accuracy combined (mean of online and offline percentages)	63.2% (24.7%)	69.6% (20.7%)	67.3% (23.6%)		0.67	0.717
Letter *n*-back accuracy combined (mean of online and offline percentages)	71.0% (29.8%)	77.4% (22.1%)	76.4% (26.3%)		0.17	0.92
Composite *z*-score (full battery)	−0.25 (0.71)	0.13 (0.82)	0.11 (0.81)	12.85		0.0002

The composite *z*-scores (online) were −0.25 (SD 0.74) for sham stimulation, 0.15 (SD 0.87) for left anodal stimulation, and 0.09 (SD 0.81) for right anodal stimulation, which repeated measures ANOVA showed to be significantly different (*F*_2,22_ = 7.68, *p* = 0.003). Post hoc, paired-sample *t* tests revealed that performances as measured by composite *z*-scores of the four working memory tests given during both left anodal active stimulation (*t*_11_ = 3.9, *p* = 0.003, Cohen’s *d*_*s*_ = 0.52) and right anodal active stimulation (*t*_11_ = 2.7, *p* = 0.019, Cohen’s *d*_*s*_ = 0.46) were better than performances during sham stimulation. Performances during the two active stimulation conditions (*t*_11_ = 0.60, *p* = 0.563) were not significantly different.

The composite *z*-scores (offline) were −0.18 (SD 0.92) for sham stimulation, 0.09 (SD 0.77) for left anodal stimulation, and 0.09 (SD 0.93) for right anodal stimulation, which Friedman’s test showed to be significantly different (χ^2^ = 7.17, *p* = 0.028). Follow-up Wilcoxon signed-rank tests showed that after left anodal stimulation performances were not significantly better compared to after sham stimulation (*z* = 1.88, *p* = 0.06, *r* = 0.27, Cohen’s *d*_*s*_ = 0.33), but performances after right anodal stimulation were significantly better than after sham stimulation (*z* = 2.35, *p* = 0.019, *r* = 0.34, Cohen’s *d*_*s*_ = 0.30). Although performances after right anodal stimulation were marginally better than after left anodal stimulation, the difference was not significant (*z* = 1.49, *p* = 0.136).

### Secondary outcomes: individual WM measures

Mean (SD) scores for each of the five measures that made up our overall WM composite are presented in Table 1. As shown in the first row, the participants’ longest backward spatial span was 4.8 (SD 0.9) blocks during sham stimulation compared to 6.2 (SD 1.8) blocks during left anodal stimulation and 6.0 (SD 0.9) blocks during right anodal stimulation. Friedman’s test showed that these differed significantly (χ^2^ = 9.24, *p* = 0.010). Post hoc Wilcoxon signed-rank tests indicate that left anodal stimulation (*z*_11_ = 2.21, *p* = 0.027, *r* = 0.32) and right anodal stimulation (*z*_11_ = 2.39, *p* = 0.017, *r* = 0.34) were both associated with longer maximum spans than sham stimulation. Cohen’s *d*_*s*_ effect sizes were 1.03 for left anodal stimulation and 1.39 for right anodal stimulation. Performances during the two active stimulation conditions were not different (*z*_11_ = 0.30, *p* = 0.762). We examined the role of practice effects on all tests for which significant differences were found between stimulation conditions. Participants’ longest spatial span was 5.6 blocks (SD 1.2) during session 1, 5.5 blocks (SD 1.1) during session 2, and 5.9 blocks (SD 1.8) during session 3. Friedman tests showed no significant practice effects across sessions (χ^2^ = 0.05, *p* = 0.973).

Also as shown in Table 1, participants’ longest backward digit span averaged 5.3 (SD 1.5) digits during sham compared to 5.5 (SD 1.6) digits during left anodal and 5.4 (SD 2.0) during right anodal stimulation. Friedman’s test showed no significant differences in longest digit span between conditions (χ^2^ = 0.41, *p* = 0.814).

Spatial *n*-back accuracy was calculated as (hits + correct negatives)/(total items). Online *n*-back accuracy was 62.4% (SD 25.0%) during sham stimulation, 68.6% (SD 22.1%) during left anodal stimulation, and 66.7% (SD 23.2%) during right anodal stimulation. Friedman’s test showed no significant differences between conditions (χ^2^ = 3.17, *p* = 0.205). Offline spatial *n*-back accuracy was 63.9% (SD 24.9%) after sham stimulation, 70.6% (SD 20.9%) after left anodal stimulation, and 67.7% (SD 24.2%) after right anodal stimulation, which were not significantly different (χ^2^ = 0.55, *p* = 0.758).

Letter *n*-back accuracy was calculated as (hits + correct negatives)/(total items). Online *n*-back accuracy was 69.6% (SD 30.2%) during sham stimulation, 76.2% (SD 22.5%) during left anodal stimulation, and 76.3% (SD 26.8%) during right anodal stimulation. These were not significantly different (χ^2^ = 0.5, *p* = 0.779). Offline letter *n*-back accuracy was 72.3% (SD 29.8%) after sham stimulation, 78.5% (SD 22.5%) after left anodal stimulation, and 76.5% (SD 25.9%) after right anodal stimulation. These also were not significantly different (χ^2^ = 0.17, *p* = 0.920).

Brief Test of Attention (BTA) raw scores averaged 6.8 (SD 2.5) after sham stimulation compared to 7.4 (SD 2.2) after left anodal and 7.9 (SD 2.5) after right anodal stimulation. We found the predicted main effect of condition on BTA performance with Freidman’s test (χ^2^ = 7.09, *p* = 0.029). Follow-up Wilcoxon signed-rank tests revealed that BTA performance after left anodal stimulation was not significantly different from BTA performance after sham stimulation (*z*_11_ = 1.63, *p* = 0.102, *r* = 0.24, Cohen’s *d*_*s*_ = 0.27), but BTA performance after right anodal stimulation was better than after sham (*z*_11_ = 2.26, *p* = 0.024, *r* = 0.33, Cohen’s *d*_*s*_ = 0.46). Additionally, BTA performance was better after right than left anodal stimulation (*z*_11_ = 2.45, *p* = 0.014, *r* = 0.35, Cohen’s *d*_*s*_ = 0.22). There was no effect of practice on BTA performance from session 1 to session 3 (*z*_11_ = −1.12, *p* = 0.265). The results of our secondary analyses are depicted in Fig. 3a–c.

### Blinding

We hypothesized that participants would tolerate tDCS and remain blind to condition. All participants completed the three sessions with no adverse events. After each session, all 12 participants were asked to indicate (yes/no) whether they thought they had received real stimulation. Eleven participants endorsed left anodal stimulation as real, nine endorsed right anodal stimulation as real, and 10 endorsed sham as real. McNemar’s test of related samples showed no differences in endorsement rates between sham and both left (*p* > 0.99) and right (*p* > 0.99) active anodal stimulation.

## Discussion

In this study, the effects of tDCS on some WM tasks are among the largest in the literature. Meta-analyses of attempts to enhance working memory have typically shown smaller enhancing effects of anodal stimulation at F3 in both healthy adults and clinical populations [61, 62]. We observed a Cohen’s *d*_*s*_ of 0.50 for full-battery WM performance (as measured by composite *z*-scores) associated with left anodal stimulation compared to sham stimulation and 0.47 for right anodal stimulation compared to sham stimulation. The measures on which we observed the largest significant effects of tDCS were backward spatial span (*d*_*s*_ = 1.33) and the Brief Test of Attention (*d*_*s*_ = 0.46). These effect sizes are similar in magnitude to those reported in other investigations of balanced bifrontal stimulation to enhance WM [33, 39–42]. Although enhanced performance was not significant on some individual WM tasks in the battery, group performance associated with either type of active stimulation was at least equal to and in most cases, better than sham on all tasks (see Fig. 3a) and most of the participants showed improved composite *z*-scores in both active conditions (see Fig. 3d).

The larger than typical effect sizes reported here could be attributed to several factors. WM deficits in individuals with ASD might provide more room for improvement than in healthy adults. Also, we used a balanced bifrontal montage, which not only capitalizes on the direct effects of anodal stimulation but also likely combines them with the effects of contralateral disinhibition, as well as a tendency to recruit additional aspects of working memory networks [57]. Additionally, stimulation was delivered during WM performance, which seems to produce the most consistent effects [61]. Finally, our participants received active tDCS at a current density of 0.06 mA/cm^2^ for 40 min. This is twice as long as it has been typical of behavioral studies, although periods of 30 min and longer [32, 38, 40, 41, 79–83] are becoming common in recent years, especially in studies of cognition. Longer stimulation periods seem to more effectively modulate WM [62], current density greater than 0.029 mA/cm^2^ is positively correlated with greater effects on WM [62], and total charge delivered is positively correlated with effect size in studies of cognition [78].

**Fig. 4:**
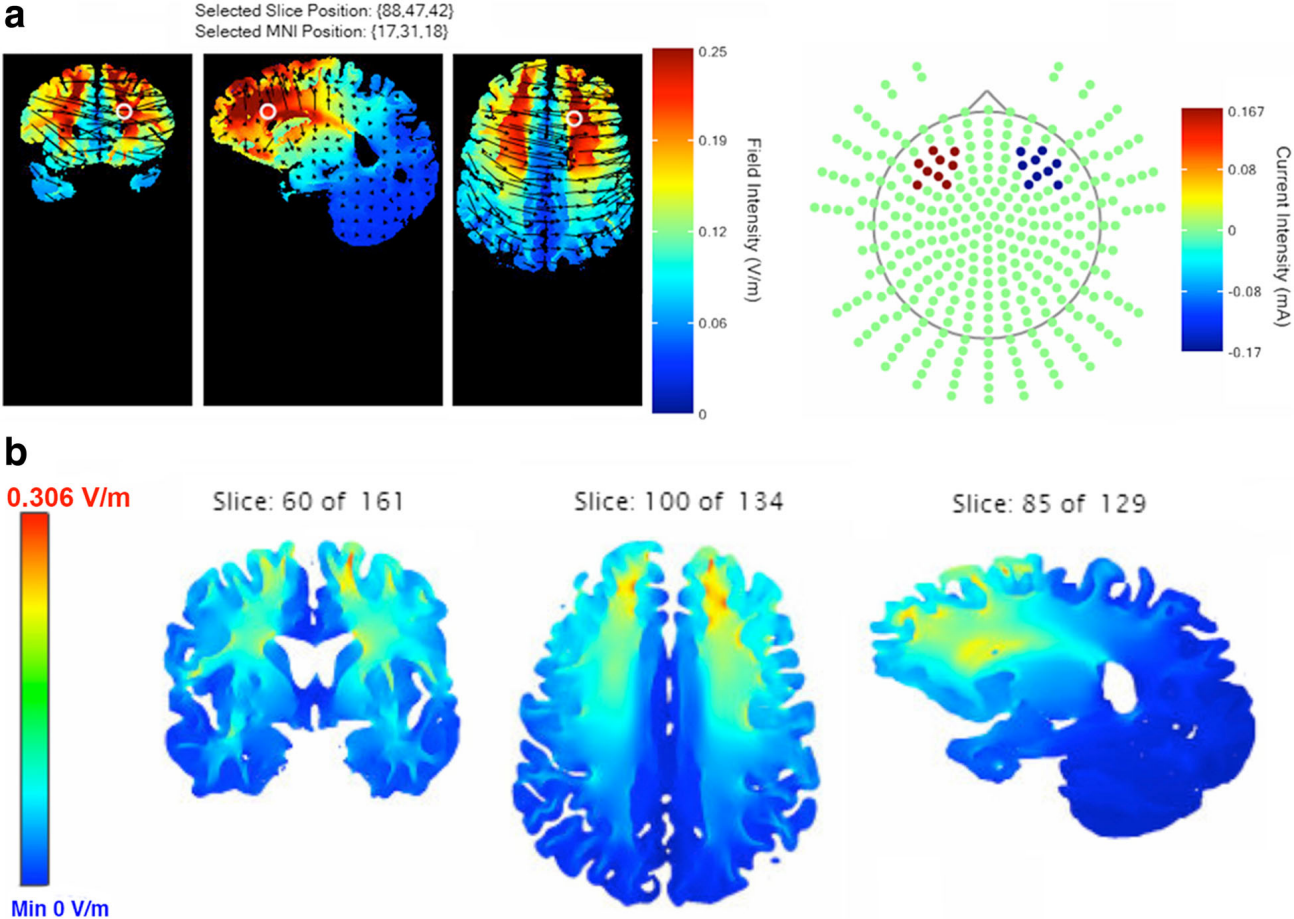
**a** Simulation of 1.0 mA current flow with an F3 anodal-F4 cathodal montage from Soterix TDCS Explore. Maximum field intensity is 0.25 V/m. Current is disbursed throughout the frontal cortex. **b** Simulation of 1.0 mA current flow with an F3 anodal-F4 cathodal montage from http://neuralengr.com/bonsai/. Maximum field intensity is 0.306 V/m. Current flows throughout frontal cortex and penetrates to subcortical areas

Two characteristics of tDCS are particularly intriguing when considering its potential for cognitive enhancement: (1) its functional resolution and (2) its broad distribution of current. Functional resolution is the tendency of tDCS to alter synaptic plasticity preferentially in networks that are undergoing task-related activity during stimulation [84–86]. Broad distribution of current has been demonstrated via electroencephalography [87, 88], fMRI [28, 89–92], and positron emission tomography [92], all of which have been used to show that tDCS alters cortical activity both beneath the electrodes and in more remote regions. Our two current flow simulations suggested that with the balanced bifrontal montage, current flow is not limited to the DLPFC, but is broadly distributed to prefrontal cortical regions as well as to subcortical regions via white matter tracts (see Fig. 4). Wide current distribution and functional resolution could be particularly advantageous for WM enhancement given the broad topology of WM networks and heterogeneous etiologies of WM dysfunction.

The broadly distributed current and functional resolution of tDCS prompt us to consider several possible mechanisms of action rather than restricting ourselves to the oversimplified view that the enhancement of WM found in this study is due solely to improved maintenance and manipulation of information driven by neuromodulation of the DLPFC.

TDCS also may have increased attentional control of WM by improving top-down signaling from the prefrontal cortex. Functional MRI studies of healthy adults show a diffuse WM network characterized by core attentional control nodes in bilateral DLPFC [93]. When WM demand is low, individuals with ASD and typically developing controls both rely on bottom-up parietal processes to direct external attention. As loads increase, prefrontal activity increases in typically developing controls but not persons with ASD, suggesting a failure to exert sufficient top-down control of attentional resources [49]. In ASD, reduced anterior-posterior connectivity [44, 45] likely contributes to the PFC’s poor integration with the rest of the WM network. Anodal tDCS over DLPFC can strengthen anterior-posterior connectivity in dorsolateral-parietal attentional networks [28, 43], which may have temporarily improved attentional control mediated by the PFC. Also, Park and colleagues [94] showed that following a stroke, 30 min of anodal stimulation over left DLPFC enhanced subsequent performance on the Auditory Continuous Performance Test, a test of attention and response control on which individuals with ASD do poorly [95]. Our finding of enhancement on a task with both working memory and attentional components (BTA) further supports improved attentional control as a potential mechanism for performance enhancement.

Another possible benefit of tDCS could be improved emotional regulation mediated by the right DLPFC [96], which is hypoactive in ASD [46]. Better emotional control could help participants combat frustration as WM loads increase [97] and resist the effects of stress [98]. Enhanced PFC integration with the rest of the WM network may also improve the ability to suppress irrelevant information [99] and ignore interference [100].

Yet another possibility is that tDCS improves vigilance. Using the same balanced bifrontal montage, Nelson and colleagues [101] showed enhanced vigilance on an air traffic control simulator as measured by better discrimination (A’) following left anodal stimulation compared to sham. Executive control of vigilance has been linked to the right PFC for easier tasks and to bilateral PFC for more difficult tasks. Here, we found that both left and right anodal stimulation improved overall WM and that BTA performance improved after right compared to left anodal stimulation. The errorless BTA performance of a few participants, even after sham stimulation, suggests that this task was less demanding than the others, which could explain the relatively greater benefit of right vs. left anodal stimulation.

Current flow simulations and studies using fMRI [102] indicate that during balanced bifrontal stimulation, current passes through the anterior cingulate. The anterior cingulate cortex is activated for many types of executive function tasks, including vigilance, performance monitoring, and error avoidance. Individual variability in WM dysfunction and the theorized functional resolution of tDCS suggest that the same tDCS montage could strengthen response inhibition in one person, improve response monitoring in another, and improve vigilance in a third person.

Given traditional assumptions of an excitatory role for anodal stimulation and an inhibitory role for cathodal stimulation, as well as previous research suggesting right anodal stimulation of the DLPFC is less effective [61], the finding that both active bifrontal montages were similarly effective in enhancing performance may seem counter-intuitive. There is growing evidence that the canonical assumptions of anodal excitation and cathodal inhibition beneath the electrodes are often violated [53, 54]. Also, direction of current flow may begin to matter less as amperage increases from 1 to 2 mA [103], which would suggest that the montages are more equivalent in their effects at areas more remote from the electrodes, such as the anterior cingulate. Several of the potential mechanisms for enhancement described in the preceding paragraphs could be triggered by either left anodal stimulation or right anodal stimulation. For example, both bifrontal montages could have increased anterior-posterior connectivity that is typically reduced in working memory networks [44, 49, 104], and both montages would have passed current through the anterior cingulate, which is desynchronized from other aspects of working memory networks [105] and which shows abnormal activity related to attentional and executive control in individuals with ASD. Finally, right anodal stimulation also may have ameliorated the poor emotional regulation typically driven by right prefrontal hypoactivity in ASD.

The findings reported here are limited in several ways, which may inform improvements in future approaches. First, given that mean scores on the BTA approached the maximum for this test, ceiling effects might have obscured the benefit of active tDCS on BTA performance. A more difficult version of this task might yield a better estimate of effect size. Also, some higher-functioning participants showed ceiling effects on *n*-back accuracy rates, even at 2-back and 3-back, while lower-performing participants showed improvements with stimulation. Stimulation may enhance performance only when recipients are working near capacity. Thus, future investigations could titrate participants’ *n*-back load until previously specified performance criteria are met [33, 106]. It is also likely that the 50-min delay between the end of stimulation and the start of offline performances allowed for the effects of stimulation to diminish, so immediate offline effects of stimulation may be larger than those reported here. Although the 8-min practice time ensured that all outcomes were measured after sufficient stimulation had been delivered to alter synaptic plasticity [26, 107], the additive impact of tDCS duration on cognitive performance is unknown. Some studies show increasing effects with longer periods of stimulation [62, 108], while others show that longer periods of stimulation can shrink or even reverse effects [109]. Overall, task order did not predict effect size; the largest effects of stimulation were on the second task (spatial span backward) and the last task (BTA). The mean intersession interval was 6.8 (5.1) days, but for two participants, the interval was only 24 h. Although the effects of tDCS (relative to sham) on acquired motor skills and other learning have been shown to persist for months or longer with repeated training [106, 110, 111], we are not aware of any studies that show a single session of tDCS continuing to alter behavior or secondary measures of neural activity, such as regional cerebral blood flow, blood oxygenation, or neurotransmitter release, more than 24 h after stimulation ends. However, findings in the TMS literature have demonstrated hyperplasticity in individuals with ASD, such that motor cortical plasticity endures 2–3 times longer than in healthy controls [112, 113]. Although the combination of a 40-min stimulation period and ASD hyperplasticity could increase the possibility of carryover effects of active stimulation, offline effects would still not be expected to persist to the next day and affect subsequent performance. Also, with multiple active conditions, intersession intervals should ideally be standardized to at least 1 week to avoid metaplasticity that has been shown to endure for at least 24 h in individuals with ASD who receive theta-burst stimulation [113]. Similar effects have also been observed in repeated tDCS to enhance working memory, with training gains smaller when stimulation sessions are separated by a 24-h interval compared to a 3-day interval [106]. The effects of both hyperplasticity and metaplasticity could have reduced power; hyperplasticity could have carried over to subsequent sham performance and metaplasticity could have attenuated the effects of next-day stimulation. Such effects would likely diminish differences between active stimulation and sham. Although washout period did not predict changes in composite *z*-scores between sessions with the same order of conditions, the possibility of an interaction between condition and washout period cannot be precluded because of insufficient power to detect even a large effect.

Our results are novel in several ways. To our knowledge, this is the first demonstration of tDCS as a tool to enhance WM in individuals with ASD, which is important because WM deficits likely contribute to impairments of more complex cognitive processes and social behavior in those individuals. This also is the first report of tDCS-mediated enhancement of spatial WM using a balanced bifrontal montage. In addition, our administration of tDCS for 40 min is the longest reported period of bifrontal tDCS. Recent reviews suggest that spatial WM is more impaired than verbal WM in individuals with ASD [2], which may explain why we found larger effects of prefrontal tDCS on spatial WM performance than have previously been reported in healthy adults [100, 114].

Finally, we found that the beneficial effects of tDCS on subsequent performance persisted for at least 50 min after stimulation, which is the longest delay between stimulation and an offline effect of stimulation on WM-driven task performance of which we are aware. Previously, offline effects on subsequent WM task performance have been found to persist for as long as 30–40 min [32, 36, 38, 115] after stimulation. Clearly, longer follow-up monitoring is needed to assess effect persistence after stimulation and to attempt more enduring facilitation of WM with repeated sessions of stimulation and training. It is also worth noting that persisting offline effects of tDCS transferred to another test with a working memory component on which participants did not train during stimulation. This is consistent with previous findings of near transfer [29, 33, 85, 116, 117]. Future experiments should explore transfer from laboratory-based tasks to applied cognitive and social skills that enhance quality of life.

## Conclusions

We found that a balanced bifrontal tDCS montage can enhance both online and offline WM performance in adults with high-functioning autism. The largest effects were on spatial span and BTA performance. Left anodal stimulation and right anodal stimulation both enhanced WM, with effects ranging from small to large. Material-specific effects of anodal stimulation were not observed over either hemisphere. As hypothesized, online stimulation effects transferred to an untrained offline task with a working memory component, but enhancement (compared to sham) reached significance after right anodal stimulation only.

In summary, our pilot study suggests that tDCS shows promise as a method to enhance WM in adults with high-functioning ASD. Further studies are needed to replicate these effects in a larger sample and determine if repeated stimulation can produce lasting effects that transfer to real-world skills.

## Abbreviations

ADOS: Autism Diagnosis Observation Schedule
ASD: Autism spectrum disorder
BTA: Brief Test of Attention
DLPFC: Dorsolateral prefrontal cortex
fMRI: Functional magnetic resonance imaging
K-BIT2: Kaufman Brief Intelligence Test, 2nd Edition
PFC: Prefrontal cortex
tDCS: Transcranial direct current stimulation
WM: Working memory
WMS-III: Wechsler Memory Scale, 3rd Edition
